# Natural variation of domestication-related genes contributed to latitudinal expansion and adaptation in soybean

**DOI:** 10.1186/s12870-024-05382-0

**Published:** 2024-07-09

**Authors:** Jing Li, Yecheng Li, Kwadwo Gyapong Agyenim-Boateng, Abdulwahab Saliu Shaibu, Yitian Liu, Yue Feng, Jie Qi, Bin Li, Shengrui Zhang, Junming Sun

**Affiliations:** 1 The State Key Laboratory of Crop Gene Resources and Breeding, National Engineering Laboratory for Crop Molecular Breeding, Institute of Crop Sciences, Chinese Academy of Agricultural Sciences, 12 Zhongguancun South Street, Beijing, 100081 China; 2 Faculty of Agrobiology, Food and Natural Resources, Czech University of Life Sciences Prague, Prague, Czech Republic; 3 Department of Agronomy, Bayero University Kano, Kano, 3011 Nigeria

**Keywords:** Soybean, Latitudinal expansion, Domestication, Adaptation, Flowering time

## Abstract

Soybean is a major source of protein and edible oil worldwide. Originating from the Huang-Huai-Hai region, which has a temperate climate, soybean has adapted to a wide latitudinal gradient across China. However, the genetic mechanisms responsible for the widespread latitudinal adaptation in soybean, as well as the genetic basis, adaptive differentiation, and evolutionary implications of theses natural alleles, are currently lacking in comprehensive understanding. In this study, we examined the genetic variations of fourteen major gene loci controlling flowering and maturity in 103 wild species, 1048 landraces, and 1747 cultivated species. We found that E1, E3, FT2a, J, Tof11, Tof16, and Tof18 were favoured during soybean improvement and selection, which explained 75.5% of the flowering time phenotypic variation. These genetic variation was significantly associated with differences in latitude via the LFMM algorithm. Haplotype network and geographic distribution analysis suggested that gene combinations were associated with flowering time diversity contributed to the expansion of soybean, with more HapA clustering together when soybean moved to latitudes beyond 35°N. The geographical evolution model was developed to accurately predict the suitable planting zone for soybean varieties. Collectively, by integrating knowledge from genomics and haplotype classification, it was revealed that distinct gene combinations improve the adaptation of cultivated soybeans to different latitudes. This study provides insight into the genetic basis underlying the environmental adaptation of soybean accessions, which could contribute to a better understanding of the domestication history of soybean and facilitate soybean climate-smart molecular breeding for various environments.

**Supplementary Information:**

The online version contains supplementary material available at 10.1186/s12870-024-05382-0.

## Introduction

Soybean (*Glycine max* (L.) Merr.) is one of the most economically important oil and protein crops which provides over 25% of the world’s protein for food and animal feed [1]. Cultivated soybean was domesticated from its wild progenitor (*Glycine soja* Sieb. & Zucc.) 5000 years ago in temperate regions of China between 32°N and 40°N [2, 3] and is currently cultivated worldwide across a wide range of latitudes, from 53°N to 35°S [4]. Global warming has shifted crop planting zones northward over the past few decades [5], there is need for crop breeders to select for adaptability to different latitudes. However, the optimization of flowering time (FT) for the selection of soybean varieties across latitudes through traditional breeding is time consuming and labour intensive. Thus, to illustrate how combinations of QTLs and their interactions with the environment facilitate the photothermal adaptation of soybean to regions far beyond its center of origin, which will accelerate the selection of varieties with the capacity for latitudinal adaptation, supporting yield stability in the face of crop migration.

The broad ecological adaptability of soybean plants and the narrow habitat of individual soybean cultivars are balanced via genetic variation or diversification in QTL controlling flowering and maturity stages [6–10]. The quantitative trait loci controlling photoperiodism, which affect flowering and maturity, have been subjected to both artificial and natural selection pressures, which have led to variations that allow for the adaptation of soybean to a range of geographical regions. A growing number of gene variants contributing to these changes have been identified. Classical genetics has revealed that fourteen major QTL (E1, E2, E3, E4, FT2a, FT5a, J, Tof4, Tof5, Tof8, Tof11, Tof12, Tof16, and Tof18) control photoperiod-regulated flowering [6, 7, 11–16], which are required for the global adaptation of soybean. The identified genes have been studied individually or in pairs, and have been reported to function in the adaptation of soybean. However, these genes have been studied on few cultivars, providing only a partial explanation for their adaptability. This indicates that the genetic variation and molecular mechanism underlying soybean adaptation across various geographical regions remain to be fully eclucidated.

The impact of an individual gene on a specific trait is inherently constrained [17]. Studies on the molecular nature of these loci controlling photoperiod-regulated flowering have outlined their complex interactions, and have unveiled the synergistic effects that bolster soybean’s capacity to adapt across various latitude [6, 15, 18, 19]. For instance, the natural variations of FT2a, FT5a, and J combination revealed a large independent history of selection and played distinct roles as soybean spread to lower latitudes [10]. The four *E* genes including *E1*, *E2*, *E3*, *E4* have different impacts on maturity, and their allelic variations and combinations determine the diversification of soybean maturity and adaptation to different latitudes [20]. Additionally, Tof11 and Tof12 play central roles in the adaptation of soybean to high latitudes via stepwise selection during soybean domestication [8, 21]. This wealth of knowledge is essential for advancing molecular breeding strategies and for the cultivation of soybean varieties that are optimally adapted to a range of environmental conditions. Although there are common genetic threads, the crops have independently evolved distinct photoperiodic genes to facilitate latitudinal adaptation, complicating the identification of a universal mechanism for such adaptation. Unraveling the genetic diversity within FT-related genes and assessing their cumulative effects through the analysis of various haplotype combinations is pivotal for a deeper understanding of the significance of these genes across diverse soybean varieties. Gaining this insight will expedite the development of soybean varieties that are specifically tailored to the unique challenges of different geographical regions, enhancing agricultural productivity and resilience.

To gain a broader understanding of the molecular mechanisms underlying soybean adaptation and diversity across different latitudes, this study investigated fourteen FT-related genes (E1, E2, E3, E4, FT2a, FT5a, J, Tof4, Tof5, Tof8, Tof11, Tof12, Tof16, and Tof18). In this study, we performed the following: (i) analyzed the genetic diversity, neutrality, and effects of individual haplotypes on flowering time; (ii) evaluated the effect of FT-related genes on flowering time via genomic selection; (iii) investigated the effects of haplotype combinations of different FT-related genes on latitudinal adaptation and flowering time; and (iv) constructed geographical evolution models of flowering time genes and predicted the planting area for the main varieties. The results provide valuable information for further gene discovery and the pyramiding of genes in the breeding of soybeans that will be adapted to various environments.

## Materials and methods

### Plant materials and phenotyping

The study employed a natural populations of 2,898 soybean accessions from 25 countries, including 103 wild species, 1,048 landraces, and 1,747 cultivated species, with an average sequencing depth greater than 30X [22], were retrieved from the Genome Sequence Archive (GSA) and Genome Warehouse (GWH) databases in the BIG Data Center (https://bigd.big.ac.cn/gsa/index.jsp) under the accession number PRJCA002030 with Zhonghuang13 (ZH13) as the reference genome. A complete list of the accessions studied, along with their details, is available in Supporting Information Table S1. Phenotypes and genotype data are available at SoyOmics (https://ngdc.cncb.ac.cn/soyomics/index). The planting environments were Beijing in 2013 (BJ13, 40°13′ N/116°12′ E), Beijing in 2014 (BJ14, 40°13′ N/116°12′ E), Henan in 2014 (HN14, 34°76′ N/113°67′ E), and Henan in 2015 (HN15, 34°76′ N/113°67′ E), Shanxi in 2013 (SX13, 37°87′ N/112°53′ E), Shanxi in 2014 under drought and wet (SX14_Drought/_Wet, 37°87′ N/112°53′ E), Shanxi in 2015 under drought and wet (SX15_Drought/_Wet, 37°87′ N/112°53′ E). Beginning bloom date (BBD) was recorded at the R1 stage (as days from emergence to the appearance of the first open flower in 50% of the plants), full bloom date (FBD) was recorded at the R2 stage (as days from emergence to the appearance of open flower at one of the two uppermost nodes), pod maturity date (MD) was recorded at the R8 stage (as days from emergence to the time at which 95% of pods attained mature color) [23], the distribution of traits across the various environments were illustrated in Supplementary Fig. 1.

### Genomic selection

For GS, ridge regression best linear unbiased prediction (rrBLUP) [24] was applied for BBD, FBD, and MD. The rrBLUP model is a mixed effect linear model, given by

$$Y=\text{xb}+zu+e$$

where $\text{y}$ is a vector of phenotypes, $\text{x}$ is the fixed effect of the identity design matrix, $\text{b}$ is the fixed effect, $\text{z}$ is a matrix of genetic markers, $\text{u}$ is the marker effect as a random effect, and $\text{e}$ is the residual error. Under the linear mixed model context, two variance components ${\upsigma }_{\text{u}}^{2}$ and ${\sigma }_{e}^{2}$ for marker effect variance and residual error variance, respectively, were estimated. The predict abilities were assessed by Pearson's correlation coefficients between the observed and predicted values in 20 fivefold cross-validations. Missing genotype were imputed using the software Beagle version 4.0 [25]. For each cross-validation, all accessions were divided into five folds, and one fold was used as the testing set, while the other four folds were used as the training set. All the scenarios are based on the same cross-validation scheme.

### Genome-wide association study

For GWAS, a total of 1,298,608 SNPs of 2,898 soybean accessions were used for association analysis, with a minor allele frequency (MAF) > 5%, a missing rate < 10%, and heterozygosity < 10%. GWASs were performed based on an efficient mixed model using the EMMAX software package [26]. PLINK software was used to perform principal component analysis of the population, and the first five principal components were included as fixed effects [27]. The matrix of pairwise genetic distances derived from the simple matching coefficients was used as the variance‒covariance matrix of the random effects. We defined the whole-genome significance cutoff as the Bonferroni correction threshold [28, 29], the threshold was − log(0.05/total SNPs), and the genome-wide significance level for branch number was 3.85 × 10^−8^.

### Genotype-environment association

LFMM (latent factor mixed models) [30] was used to evaluate the associations between SNP genotypes (826,020) and environmental variables (latitude) in landraces and cultivated soybeans. To select the optimal number of latent factors for this analysis, we estimated admixture coefficients using sparse nonnegative matrix factorization (sNMF) [31], selecting a number of clusters (K) with the lowest cross entropy. LFMM was run with 10 repetitions, 100,000 iterations and a burn-in step of 50,000. Finally, we combined the resulting z scores across all runs and recalculated *p* values using the genomic inflation factor (λ). Following λ recalibration and subsequent correction for multiple comparisons, SNPs with a q value less than 0.05 were considered outliers.

### Gene annotation

SNP annotations of fourteen FT-related genes with the ZH13 V2 genome were carried out using ANNOVAR software [32], and SNPs were categorized as being in intergenic regions, upstream (that is, within a 2-kb region upstream of the transcription start site) or downstream (within a 2-kb region downstream of the transcription termination site) regions, in exons or introns. SNPs in coding sequences were further classified as synonymous SNPs or nonsynonymous SNPs. Indels in exons were classified according to whether they led to a frameshift effect.

### Genetic diversity analysis

SNP and indel data were used for genetic diversity analysis. SNPs with > 10% missing data or with a minor allele frequency (MAF) < 5% were filtered out, and indels with a maximum length of 10 bp were included. The nucleotide diversity, θ and π (number of parsimony informative sites) [33] for the wild, landrace, and cultivar populations of soybean were calculated by DNAsp v5 software to estimate the degree of variability [34].

### Molecular evolution analysis

Tajima’s D and Fu and Li’s D* F* tests were conducted to identify related genes within the population and to determine whether the coding region sequence is subject to neutral selection. The number of shared and unique mutations between the wild and domesticated populations within each gene pool was also computed as a measure of divergence. All of these estimates were obtained using DNASP, version 5.10.01 [34]. The pairwise genetic differentiation (*Fst*) [35] permutation test with 1000 replicates was computed to investigate the degree of differentiation between the wild and domesticated populations of the different gene pools by VCFtools (v0.1.14) [36]. The first 5% value was used as the threshold for the whole genome.

### Linkage disequilibrium analysis

The squared correlation coefficient (r^2^) between pairwise SNPs was computed using the software PLINK (v1.9) to estimate and compare the pattern of LD with the parameters (-ld-window-r^2^ 0 -ld-window 99,999 -ld-window-kb 1000) [27].

### Haplotype analysis of genes in the soybean population

Haplotype analysis was performed on the sequencing data using DnaSP v5 software. The frequency distribution of haplotypes was calculated by using winAr35 software [37], and the haplotype network diagrams were constructed by using the median-joining network algorithm in PopART software [38].

## Results

### Nucleotide variation and neutrality tests

The analyses of the retrieved sequences of SNPs and indels for the 14 FT-related genes from the 2,898 accessions revealed that the total lengths of the aligned coding regions for E1, E2, E3, E4, FT2a, FT5a, J, Tof4, Tof5, Tof8, Tof11, Tof12, Tof16, and Tof18 were 524, 21,613, 6343, 5754, 5187, 1786, 5406, 521, 10,127, 3445, 22,424, 14,913, 13,900, and 17,449 bp, respectively. The polymorphic sites of all 14 loci are shown in Supplementary Fig. 2. The species-wide levels of variation in SNPs varied from 1 (E1) to 298 (Tof18), and the number of indels ranged from 0 (Tof4) to 42 (Tof18) in the soybean accessions (Supplementary Table 2).

The θ [39] and π [40] values were calculated to describe the nucleotide variation in the population. The value of θ was lower in *G. max* than in *G*. *soja* (Table 1) for the fourteen FT-related genes, which is due to the loss of some rare SNPs during domestication potentially attributable to bottleneck effects. The π values at the E1 and E3 loci were higher in *G. max* than in *G*. *soja* (Table 1), which contradicts theoretical observations of significantly decreased genetic diversity owing to genetic bottlenecks for the whole genome (2.94 × 10^–3^ in *G*. *soja*, 1.40 × 10^–3^ in landraces and 1.05 × 10^–3^ in cultivars) [41]. This contradiction is due to the much higher nucleotide diversity in the coding regions of E1 and E3 in landraces and cultivars, where nonsynonymous mutations have occurred and spread to moderate frequencies in the population. There was a rapid decrease in nucleotide diversity for E2, E4, FT5a, Tof4, Tof8, and Tof12. For instance, the diversity of E4 decreased from 0.31 in *G*. *soja* to 0.02 in landraces and 0.01 in cultivars (Table 1). This observation indicates that deleterious variants in the six genes have undergone strong genetic bottlenecks, resulting in a notably distinct distribution of these variants (Supplementary Fig. 2).

**Table 1:** Nucleotide diversities per base pair × 10^3^ and statistics for neutrality tests at FT-related genes of soybean

Gene	Population	SNPs&diversity				Statistics for Neutrality Tests		
		N	θ(sequence)	θ(site)	π(site)	Tajima’s D	Fu and Li’s D*	Fu and Li’s F*
E1	Wild	1	0.19	0.19	0.06	-0.8	0.49	0.12
	Landrace	1	0.13	0.13	0.31	1.06	0.39	0.73
	Cultivar	1	0.12	0.12	0.48	2.22	0.38	1.19
E2	Wild	110	20.74	0.19	0.29	1.73	2.33**	2.49**
	Landrace	114	10.22	0.09	0.15	1.96	-1.60	0.26
	Cultivar	111	11.56	0.10	0.16	1.42	-4.06**	-1.39
E3	Wild	11	1.92	0.18	0.18	0.05	1.37	1.08
	Landrace	11	1.2	0.11	0.3	3.36**	1.15	2.41**
	Cultivar	11	1.12	0.1	0.27	3.06	1.11	2.27**
E4	Wild	16	2.88	0.18	0.31	1.99	1.59*	2.06**
	Landrace	15	1.73	0.12	0.02	-1.85*	1.36	0.13
	Cultivar	15	1.74	0.12	0.01	-1.88	-1.78	-2.24
FT2a	Wild	31	5.38	0.17	0.32	2.52*	1.56*	2.31**
	Landrace	31	4.12	0.13	0.23	1.87	1.03	1.72
	Cultivar	31	3.85	0.12	0.12	-0.18	0.44	0.2
FT5a	Wild	5	0.77	0.15	0.23	0.97	0.94	1.12
	Landrace	5	0.53	0.11	0.07	-0.53	0.78	0.39
	Cultivar	5	0.62	0.12	0.12	-0.06	-0.51	-0.43
J	Wild	17	2.88	0.17	0.3	2.12*	1.59*	2.12**
	Landrace	16	2.12	0.13	0.14	0.09	-0.62	-0.41
	Cultivar	16	1.99	0.12	0.16	0.69	1.46	1.41
Tof4	Wild	2	0.38	0.19	1.34	2.30*	0.68	1.38
	Landrace	2	0.27	0.13	0.02	-0.96	0.55	0.06
	Cultivar	2	0.25	0.12	0.01	-0.97	0.53	0.03
Tof5	Wild	72	8.83	0.18	0.69	3.11**	2.15**	3.05**
	Landrace	72	6.37	0.13	0.33	1.90	2.39**	2.62**
	Cultivar	72	5.97	0.12	0.20	0.70	0.78	0.91
Tof8	Wild	17	2.69	0.18	0.41	1.33	0.98	1.32
	Landrace	17	1.99	0.13	0.24	0.77	0.73	0.91
	Cultivar	17	1.87	0.12	0.32	1.74	0.66	1.34
Tof11	Wild	113	17.28	0.17	0.52	2.46*	2.26**	2.81**
	Landrace	113	13.15	0.13	0.47	3.25**	1.21	2.73**
	Cultivar	113	11.32	0.11	0.12	-0.21	3.02**	1.59
Tof12	Wild	43	6.72	0.18	0.45	1.70	1.43	1.84*
	Landrace	43	3.19	0.09	0.08	-0.34	1.23	0.68
	Cultivar	43	3.73	0.10	0.01	-2.21	-3.78**	-3.79**
Tof16	Wild	66	8.45	0.18	0.56	2.48*	2.13**	2.72**
	Landrace	66	6.24	0.12	0.55	4.03	2.37**	3.85**
	Cultivar	66	5.84	0.12	0.52	3.78	2.35**	3.72**
Tof18	Wild	340	56.27	0.19	0.82	3.64***	2.64**	3.70**
	Landrace	340	39.57	0.13	0.82	5.64***	4.02**	5.78**
	Cultivar	340	37.05	0.12	0.68	5.29	4.23**	5.63**

N, number of variations used for the analysis

^*^P < 0.05

**P < 0.01

***P < 0.001

To distinguish molecular variation that is neutral from variation subject to selection in molecular population genetics [42], neutrality tests, such as Tajima's D, Fu and Li's D*, and Fu and Li's F* [43–45], which are commonly used to identify candidate genes under selection, were conducted to describe the population genetic characteristics of the fourteen genes. There were significant and positive Tajima's D values in E3, E4, FT2a, J, Tof11, Tof16, and Tof18, which show that those genes were under strong selection (Table 1). And the average LD values of 2 megabase (Mb) genomic regions spanning the genes in the three diversity panels were consistant with it (Supplementary Fig. 3). Meanwhile, the values of Tajima's D, Fu and Li's D*, and Fu and Li's F* of E2, E4, FT5a, Tof4, and Tof12 shifted from positive values in *G*. *soja* to considerably negative values in *G. max.* This indicates that the variation of these might be deleterious and under positive selection in wild soybean in the natural environment, but they are favored by humans and subjected to negative selection in domesticated soybean.

### Comparative evaluation of the predictive capacity of beginning bloom date

**Fig. 1:**
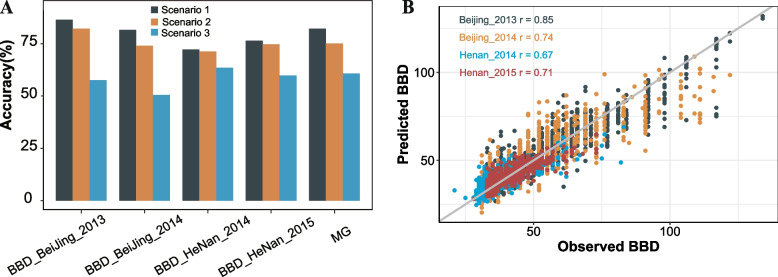
Comparative evaluation of predictive performance for beginning bloom date (BBD). **A** The predictions for beginning bloom date and maturity group are performed using three distinct scenarios: genome-wide SNPs (Scenario 1), flowering time (FT)-related genes (Scenario 2), and significant SNPs related to BBD (Scenario 3). The prediction accuracies are measured by the mean values of Pearson correlation coefficient between measure and predicted beginning bloom date with 100 cross-validations (i.e. 20 repetitions of fivefold cross-validation). **B** The performance prediction of beginning bloom date with FT genes in each individual environment. The diagonal line indicates the exact match between observed and predicted values

To determine whether the cloned FT-related gene could explain a majority of the phenotypic variance in BBD, we examined three scenarios for genomic prediction based on rrBLUP models. Scenario one included all SNPs, termed genome-wide SNPs (1,298,608); scenario two included SNPs that fall in sequences of FT-related genes, termed FT SNPs (300) (Supplementary Table 3); and scenario three comprised of significant SNPs related to BBD (19, 82, 161, 90 for BJ13, BJ14, HN14, HN15) (Supplementary Table 4 and Supplementary Fig. 5–9). It is worth noting that scenario 2 explained 82.17%, 74.05%, 71.19%, and 74.70% of the flowering time variations in BJ13, BJ14, HN14, and HN15, respectively (Fig. 1B). This was slightly lower than that in scenario 1, which explained 86.47%, 81.55%, 72.21%, and 76.36%, respectively, similar results could be found in other environments and other trait (Supplementary Fig. 4). On the hand, scenario 3 explained the lowest flowering time variations (57.58%, 50.48%, 63.49%, and 59.72%) (Fig. 1A). Obviously, scenarios 1 and 2 outperformed scenario 3 in all four environments. The highest prediction accuracy for scenario 1 was 86.47% in Beijing 2013, scenario 2 was slightly lower than scenario 1, with the difference between them ranging from 1.0% to 7.5%, similar patterns were observed in full bloom date (FBD), maturity date (MD), and maturity group (MG) (Supplementary Table 6 and Supplementary Fig. 4). Moreover, significant SNPs (scenario 3) could explain 63.49% of the phenotypic variance in Henan 2014, and only 50.48% of the variance in Beijing 2014. When all genome variations are combined, BBD performance increased from 0.58 to 0.86 (Beijing 2013), 0.50 to 0.82 (Beijing 2014), 0.63 to 0.72 (Henan 2014), and 0.60 to 0.76 (Henan 2015). This indicates that whole-genome variation is effective for predicting genomic selection, and the FT-related genes could explain the majority of the phenotypic variance in BBD. Taken together, there were similar results using genome-wide SNPs and FT-related SNPs, which show that FT-related SNPs identified in our study are representative markers. This suggests that the FT-related SNPs in the study are relatively robust to the confounding effects of geographical factors and are largely shaped by environmental gradients across accessions.

### Haplotype distribution with latitude

**Fig. 2:**
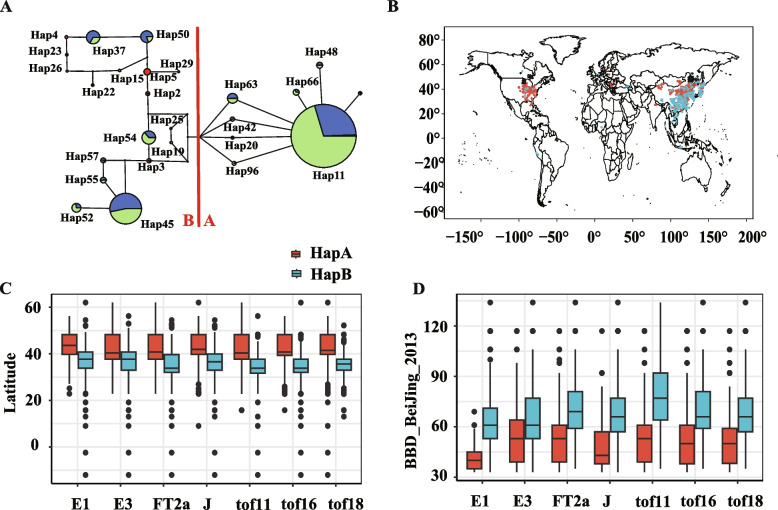
Relationships among haplotypes and the geographical distribution patterns of FT-related genes in soybean. **A** Allele network analysis for FT2a. Allele frequencies are proportional to circle size. The proportions of the three soybeans (wild soybean, landrace, and cultivar) are represented by red, blue, and green, respectively. **B** Geographical distribution of HapA and HapB for FT2a in the world. Each circle represents one accession. **C** Comparison of latitude between accessions with HapA and HapB for E1, E3, FT2a, J, Tof11, Tof16, and Tof18, respectively. **D** Comparison of beginning bloom date in Beijing 2013 between accessions with HapA and HapB. The red and blue bars represent northward (HapA) and southward (HapB) alleles of E1, E3, FT2a, J, Tof11, Tof16, and Tof18, respectively. *P* values were produced by two-tailed t-tests and Wilcoxon signed-rank tests

To explore the latitudinal adaptation of FT-related genes, we compared the relationships between FT-related gene haplotypes across latitudes. Through an integrative approach, encompassing a comprehensive analysis of haplotype frequency distribution, genetic distance analysis, and phylogenetic relationships, two haplotype groups, names as HapA and HapB were found (Fig. 2A and 2B). The gene with a single haplotype group predominating by more than 80% of the accessions will be ignored. E2, E4, FT5a, Tof4, Tof5, Tof8 and Tof12 differed geographically with pretty low haplotype diversities, and their high-frequency haplotypes accounted for 84%, 99%, 99%, 99%, 96%, 83% and 92% of the 2,898 accessions, respectively. For example, accessions with HapB of E4 and FT5a were distributed across nearly all soybean cultivation regions from 23°S to 45°N.

There was significant difference (*P* < 0.05) between the latitudes of HapA and HapB across genes. Soybean accessions carrying HapA exhibited a higher average latitude compared to those with HapB, as follows, E1 (43°69′ N and 38°11' N), E3 (41°54′ N and 37°11' N), FT2a (41°73' N and 35°02' N), J (42°76' N and 36°50' N), Tof11 (41°46' N and 33°96' N), Tof16 (41°90' N and 34°39' N), and Tof18 (42°38' N and 35°32' N) (Fig. 2C). Correspondingly, the BBD of Beijing 2013 for accessions carrying HapA was significantly earlier than those with HapB. The average BBDs for accessions with HapA of E1, E3, FT2a, J, Tof11, Tof16, and Tof18 were 41.29, 53.88, 53.13, 47.22, 52.52, 52.21, and 51.83 days, respectively. On the contrary, accessions with HapB of the same genes had average BBD’s of 63.66, 65.31, 72.30, 67.98, 75.78, 70.91, and 68.53 days (Fig. 2D). The geographical distribution of samples from both haplotype groups showed that the frequency of HapB of the seven FT-related genes gradually increased when moving southward. Accessions with HapA were found at the greatest frequency in high-latitude regions, including Heilongjiang, Jilin, Liaoning, Shanxi, Beijing, and Tianjin in China, as well as Japan and South Korea. Accessions with HapB were found at the greatest frequency, mainly in the southern regions of China, as well as in tropical and subtropical regions of South and Southeast Asia (Fig. 2B, 2C and Supplementary Table 1).

**Fig. 3:**
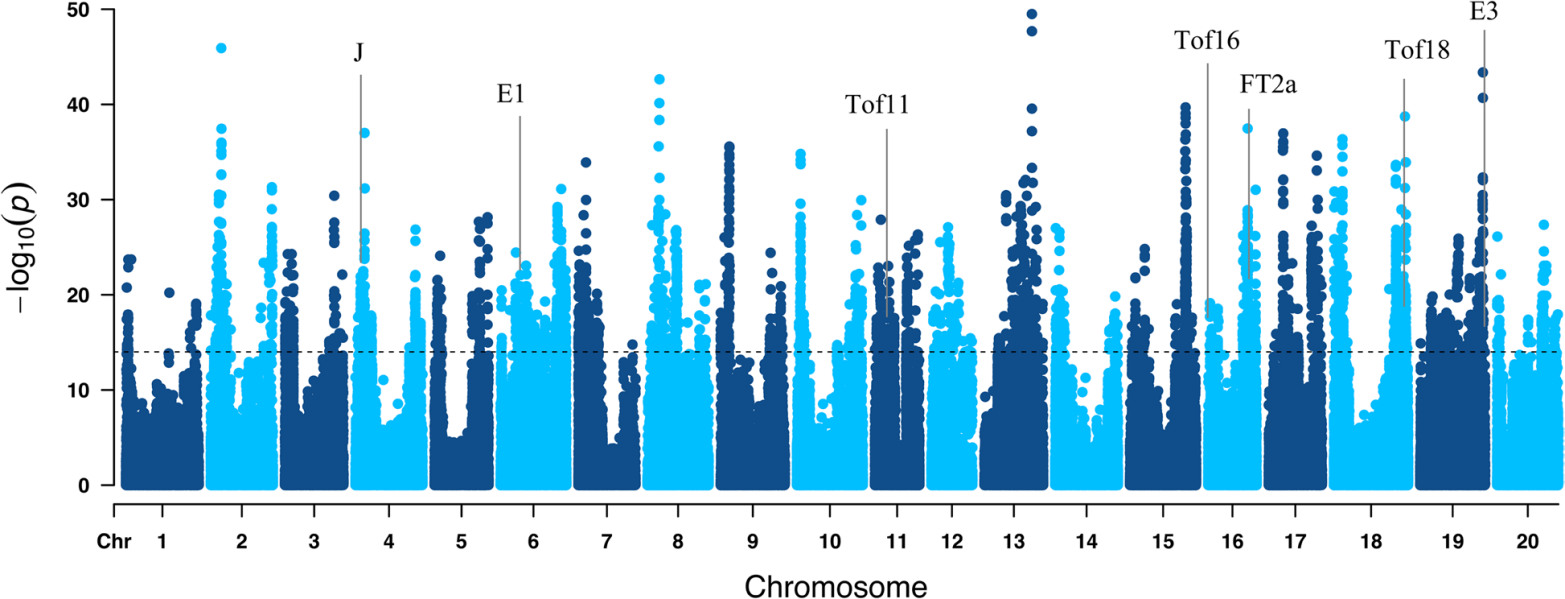
Genome-wide screening of the loci associated with local environmental adaptation. Manhattan plot for variants associated with latitude. FT-related genes are labeled in the plot at their respective genomic positions. Grey vertical lines correspond to the positions of genes. The significance threshold indicated by horizontal dotted lines

To support this observation and further investigate whether E1, E3, FT2a, J, Tof11, Tof16, and Tof18 are associated with latitude, a latent factor mixed model (LFMM) analysis, an effective algorithm for testing gene‒environment associations, was conducted (Supplementary Fig. 10). From the analysis, strong signals near seven FT-related genes were identified (Fig. 3; Supplementary Table 5), suggesting that seven FT-related genes might be associated with latitude in landraces. Collectively, these results revealed that E1 (Gm06.20204717, 1.03E-20), E3 (Gm19.47698060, 5.12E-10), FT2a (Gm16.31118627, 1.32E-13), J (Gm04.4072629, 4.55E-22), Tof11 (Gm11.11256564, 6.22E-20), Tof16 (Gm16.1533272, 1.06E-13), and Tof18 (Gm18.51220722, 1.27E-10) may play important roles in the latitudinal adaptation of soybean. Taken together, our findings indicate that E1, E3, FT2a, J, Tof11, Tof16, and Tof18 exhibited strong associations with latitude and predominantly accumulated along latitudes. As a result, for further studies, we focused on E1, E3, FT2a, J, Tof11, Tof16, and Tof18.

### Geographical evolution models of the genes with local adaptation

To explain the genetic basis of the adaptation of different soybean varieties to different ecological regions, we analyzed the geographical distribution of haplotype combinations of the seven FT-related genes (Table 2). A geographic distribution analysis of 2,898 soybean accessions showed that a major proportion of haplotype combinations had a trend of increasing along higher latitudes. A meticulous enumeration of HapA occurrences within the soybean accessions was performed, resulting in a stratification into discrete "classes". This classification system, ranging from 0A, signifying the absence of HapA, to 7A, denoting the maximum presence of HapA in an accession, provides a clear framework for genetic analysis.

**Table 2:** The distribution of E1-E3-FT2a-J-Tof11-Tof16-Tof18 genes combination of soybean

Classes	No. of varieties			
	Wild	Landrace	Cultivar	Total
0A	6	20	52	78
1A	5	65	92	162
2A	4	129	112	245
3A	4	110	119	233
4A	16	146	93	255
5A	5	218	92	315
6A	1	379	102	482
7A	1	369	47	417
Total	42	1436	709	2187

**Fig. 4:**
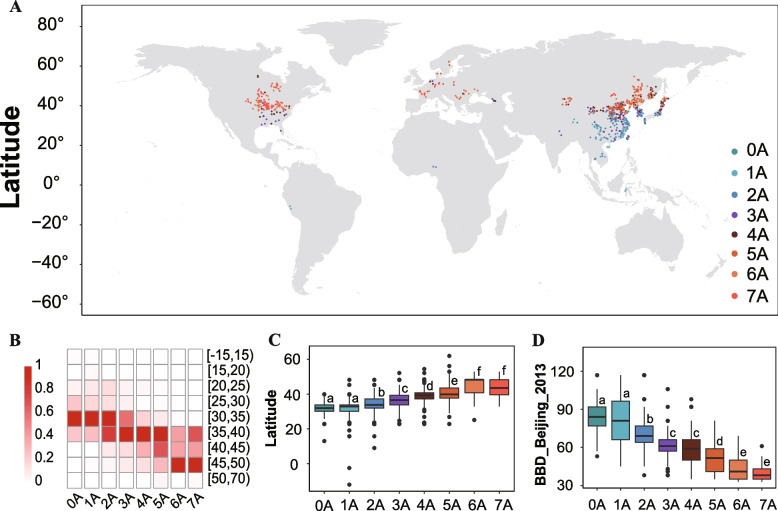
The geographical distribution of haplotype combinations and the relationship between haplotype combination and latitude. **A** Geographical distribution of haplotype combinations of seven FT-related genes on the map of the World. Haplotype combinations containing between zero and seven HapAs are indicated by dots of different colors. **B** Frequency distributions of the gene combination along latitudinal gradients in the world. **C** Comparison of latitude distribution for samples with different haplotype combinations of seven FT-related genes. **D** Comparison of beginning bloom date distribution for samples in Beijing 2013 with different haplotype combinations of seven FT-related genes. Haplotype combinations containing between zero and seven HapAs are shown on the x-axis. Letters above the bars are ranked by the Duncan test at *P* < 0.05; different letters indicate significant differences

While the 0A/1A type was present mainly in relatively low latitudes with relatively high temperatures and the 6A/7A type was present in relatively high latitudes with relatively low temperatures, implying regional differentiation in FT-related genes (Fig. 4A and Supplementary Table 1). Four hundred and seventeen (417) accessions contained all seven HapAs and accounted for 19.07% of all individuals. The accessions comprised one wild soybean, 369 landraces, and 47 cultivars. Four hundred and eighty-two accessions (1 wild soybean, 379 landraces, and 102 cultivars) had six HapAs and accounted for 22.04% of all individuals. These two classes were mainly distributed in Heilongjiang, Jilin, Liaoning, Inner Mongolia, Liaoning, Illinois, and Ontario. Eight hundred and three samples had three, four, and five HapAs, which accounted for 36.72% of all individuals; 64.50% of individuals in 35°N-40°N in China and 53.18% of individuals in 35°N-40°N in the World. These individuals were distributed in the Huang-huai-hai region, including Henan, Shandong, Beijing, and Shanxi. Two hundred and forty-five accessions (4 wilds, 129 landraces, and 112 cultivars) had two HapAs which accounted for 11.20% of all individuals, and were mainly distributed in Beijing, Shandong, and Shanxi. One hundred and sixty-two samples (5 wilds, 65 landraces, and 92 cultivars) had one HapA, accounted for 7.4% of all individuals and were mainly distributed in Henan, Jiangsu, Hebei, and Shandong. Seventy-eight samples had all seven HapB and accounted for 3.57% of all individuals. The samples comprised of 6 wild soybeans, 20 landraces, and 52 cultivars, and they were mainly distributed in Jiangsu, Sichuan, and Anhui (Table 2, Supplementary Table 7 and 8). These results indicate that soybean accessions containing more than five genes with HapA can be grown in Northern regions. As cropping regions move Southward, a greater number of haplotypes fall into HapB.

### Validation of the geographical evolution models

The validation showed that the frequency distributions of gene combinations vary significantly across different latitudes (Fig. 4B). Aside from the 7A haplotype, which occurs at a low frequencies at low latitudes, 0A, 1A, and 2A haplotypes occur at relatively higher frequencies at low latitudes (Fig. 4A, 4B, and 4C). Approximately 71% of the soybean accessions in southern 35°N had 0A, 1A, or 2A in China, and 70.66% had 0A, 1A, or 2A in the world. Seventy three percentage (73%) of the individuals in the northern area of 40°N had more than six HapAs (Fig. 4A and 4B). This suggests that the additive effects of dominant allelic variants of the seven identified genes are important in the adaptation of soybean accessions to latitude. Moreover, the average latitudes for the different gene combinations of the seven genes were 31°7′ N, 31°9′ N, 34°1′ N, 36°4′ N, 39°2′ N, 41°0′ N, 44°8′ N, and 44°1′ N. Significant differences were identified among 0A/1A, 2A, 3A, 4A, 5A, and 6A/7A in the soybean accessions (Fig. 4C), which indicates that gene combinations contributed to the latitudinal expansion of soybean accessions and was also required for the adaptation of soybean accessions to different latitudinal regions.

We found that the BBD was linearly negatively related to the number of haplotype A (*R* = -0.74). The BBD in Beijing 2013 for accessions categorized from 0 to 7A was 85.4, 81.5, 72.7, 62.4, 58.6, 50.9, 43.4, and 39.5 days, respectively (Fig. 4D). The accessions with the least number A haplotypes flowered later contrastry to the accessions with higher number of A haplotypes which flowered earlier (Fig. 4D). These results indicated that the flowering-related loci had a roughly additive effect. Therefore, a combination of haplotypes from different genes leads to soybean varieties with a relatively continuous flowering time. This phenotypic diversity was notably observed in Beijing 2013, where the BBD ranged from 33 to 134 days. Similar results could be found in other environments, including Beijing, Henan, and Shanxi, as well as in other related traits such as FBD and MD (Supplementary Fig. 11).

To further validate the practicality of the geographical evolution model, we applied it to predict the suitable planting zone for the main soybean cultivars in the northern, Huang-Huai-Hai, and southern regions (Supplementary Table 1). Among them, the widely cultivated varieties in the northern region, such as Heihe 43, Henong 95, and Heihe 45, have a genotype combination of 7A, which is consistent with their actual planting areas in Heilongjiang and Inner Mongolia. In the Huang-Huai-Hai region, the cultivar Jidou 12 planted in Hebei was predicted with a 4A genotype, Qihuang 34, which is suitable for planting in Henan, Hebei, and Jiangsu, has a 2A genotype. In the southern region, the cultivars Zhongdou 40 and Zhongdou 43 suitable to plant in Hubei and Anhui, with a predicted result of 0A. The representative varieties from different regions were accurately predicted for their suitable planting areas, providing valuable theoretical guidance for soybean cultivation practices.

## Discussion

The process by which plants adapt to different environments is exceedingly complex. Currently, multiple functional genes have been identified based on important varieties. Growing research has revealed that the collaborative roles of multiple genes in determining flowering time. For instance, Gao et al. demostrated the significant influence of the combinations of DTH7, Ghd7, and DTH8 on rice flowering under various agricultural environments, which could improve the adaptation of rice [46]. Soybean adaptation to different latitudes involves photoperiod insensitivity, which has emerged redundantly through multiple combinations of independently generated alleles at the E1, E3, and E4 loci [19]. Thus, alleles associated with regional adaptability should be taken into consideration for genetic improvement. Supporting this hypothesis, our findings indicate that the alleles of all seven FT-related genes independently regulate latitude expansion of soybean varieties. First of all, fourteen FT-related genes explained a majority of BBD variation, similar to the whole genome (Fig. 1A and B). Second, a clear regional distribution pattern of these alleles was observed. The distribution of the alleles E1, E3, FT2a, J, Tof11, Tof16, and Tof18 alleles affected the distribution of soybean varieties, with a clear correlation with latitude (Fig. 2C and Fig. 3). Third, the E1, E3, FT2a, J, Tof11, Tof16, and Tof18 haplotype combinations exhibited obvious geographical evolution (Fig. 4A, 4B, and 4C). The genetic diversity and neutrality tests of FT-related genes and their haplotype combinations across a broad spectrum of soybean accessions facilitates understanding into the local adaptation and expansion process of soybean. This comprehensive genetic examination not only deepens our understanding of the evolutionary processes that have shaped soybean's adaptability but also sheds light on the intricate regulatory mechanisms governing these adaptations.

Plants have successfully adapted to a wide range of regions due to the presence of various allelic combinations of a series of major maturity loci. However, the mechanisms by which these genes regulate their origin and expansion pattern are still not fully understood in soybean. Exploration of these issues will facilitate an understanding of the comprehensive role of these genes in soybean. Our study revealed the presence of both HapA and HapB haplotypes in the E1, E3, FT2a, J, Tof11, Tof16, and Tof18 genes. When the cultivated soybean area expanded northward beyond 35°N, HapA completely replaced HapB for these genes (Fig. 4A, B, and C). Taken together, our geographical analysis and network studies suggest that the alleles of these genes with regional adaptability were recently and independently. Thus, we intimate that there is a gradual process of selection acting on FT-related genes as soybean areas expand northward or southward. Elucidation of the evolutionary trajectories of these alleles as well as the relationship between them and the geographical distribution of soybean provides valuable opportunities to tailor breeding programs, aligning them with the goal of enhancing soybean’s environmental adaptability.

At present, genetic studies of soybean domestication-related genes, aside being limited to few accessions have provided limited variation information and partial explanation for the local adaptability of soybeans. Firstly, our studied reveal that the fourteen FT-related genes explained 75.5% of the variation in flowering time, comparable to the variation by whole genome analysis (~ 80%). This suggests a major role for FT-related genes, although few genes with minor effects need to be further discovered. Secondly, E1, E3, FT2a, J, Tof11, Tof16, and Tof18 collaboratively contributed towards local adaptation. The genetic relationships of these haplotypes, and the relationships between haplotype combinations and geographical distributions provide a new perspective for dissecting the genetic basis of domestication-related genes in soybean. Thirdly, the variation in coding regions in soybean FT-related genes plays an important role in the regulation of latitudinal expansion. Different from maize, which domestication more frequently favored standing, gain-of-function, and regulatory variation, the domestication of soybean is similar to rice which favours de novo, loss-of-function, and coding variation [47]. It was proven that the genetic variations in the coding regions of E1, E3, FT2a, J, Tof11, Tof16, and Tof18 significantly correlated with latitude (Fig. 2C). Meanwhile, the research shows that genotype-environment associations facilitate our understanding of molecular basis of soybean environmental adaptation (Fig. 3), as evidenced in bird, sorghum, and maize [48–50]. Our findings provide a better understanding of domestication-related genes and insights into the interaction effects of domesticated genes in soybean accessions, which is beneficial for soybean breeding using molecular techniques.

Genomic selection (GS) takes into consideration the effects of all available genetic markers for the prediction of breeding values instead of only those passing a significance threshold, which, according to the infinitesimal model, approximates the genomic underpinnings of a complex trait [51]. Our findings support this advantage, as the prediction accuracy of GS was much greater than that of BBD GWAS signals. These results are consistent with previous studies which indicated no increase or decrease in prediction accuracy for models including fixed-effect covariates tagging peak GWAS signals [52]. Interestingly, the prediction accuracy greatly improved with cloned genes indicating that major genes that explain a substantial amount of phenotypic variance and very valuable in predicting phenotypic performance. While some traits are controlled by a few major genes or many minor genes, other exhibit a polygenic nature, involving a complex mixture of large- and small-effect genes [53]. Thus, the performance of the GS model should be evaluated on a trait-by-trait basis prior to its integration into a breeding program.

Cultivating soybean varieties with broad adaptability across diverse latitudes will be critical to ensure global food security. In this study, we established geographical evolution models to assess soybean latitudinal adaptation status using FT-related genes at different latitudes. The use of these models in conventional breeding programs will accurately predict the flowering time/planting area of soybean varieties and aid in the development of cultivars for climate adaptation. Secondly, developing molecular markers for the various haplotypes of E1, E3, FT2a, J, Tof11, Tof16, and Tof18 will enable breeders directly select for genotypes with locally adapted alleles, improving soybean fitness in diverse environments. The results of this study provide new insight for identifying broadly adaptable genotype combinations. Furthermore, the geographical evolution model's predictive accuracy across various regions demonstrates its potential utility in guiding the strategic planning of soybean cultivation, offering a rapid and efficient method to accelerate latitude-adaptation selection, optimizing the use of agricultural resources, and enhancing the efficiency of soybean production, contributing to future food security and sustainable agriculture.

### Supplementary Information

Supplementary Material 1.

Supplementary Material 2.

## Data Availability

Sequence data could be retrieved from the Genome Sequence Archive (GSA) and Genome Warehouse (GWH) databases in the BIG Data Center under the accession number PRJCA002030.
