# Supplementary material for: Natural variation of domestication-related genes contributed to latitudinal expansion and adaptation in soybean
Source: BMC Plant Biol. 2024 Jul 9;24:651. doi: 10.1186/s12870-024-05382-0 (PMC11232268; doi:10.1186/s12870-024-05382-0)

Supplementary Figure 1. Boxplot for the beginning bloom date, full bloom date, and pod maturity date of 2,898 soybean accessions in Beijing, Henan, and Shanxi environments.


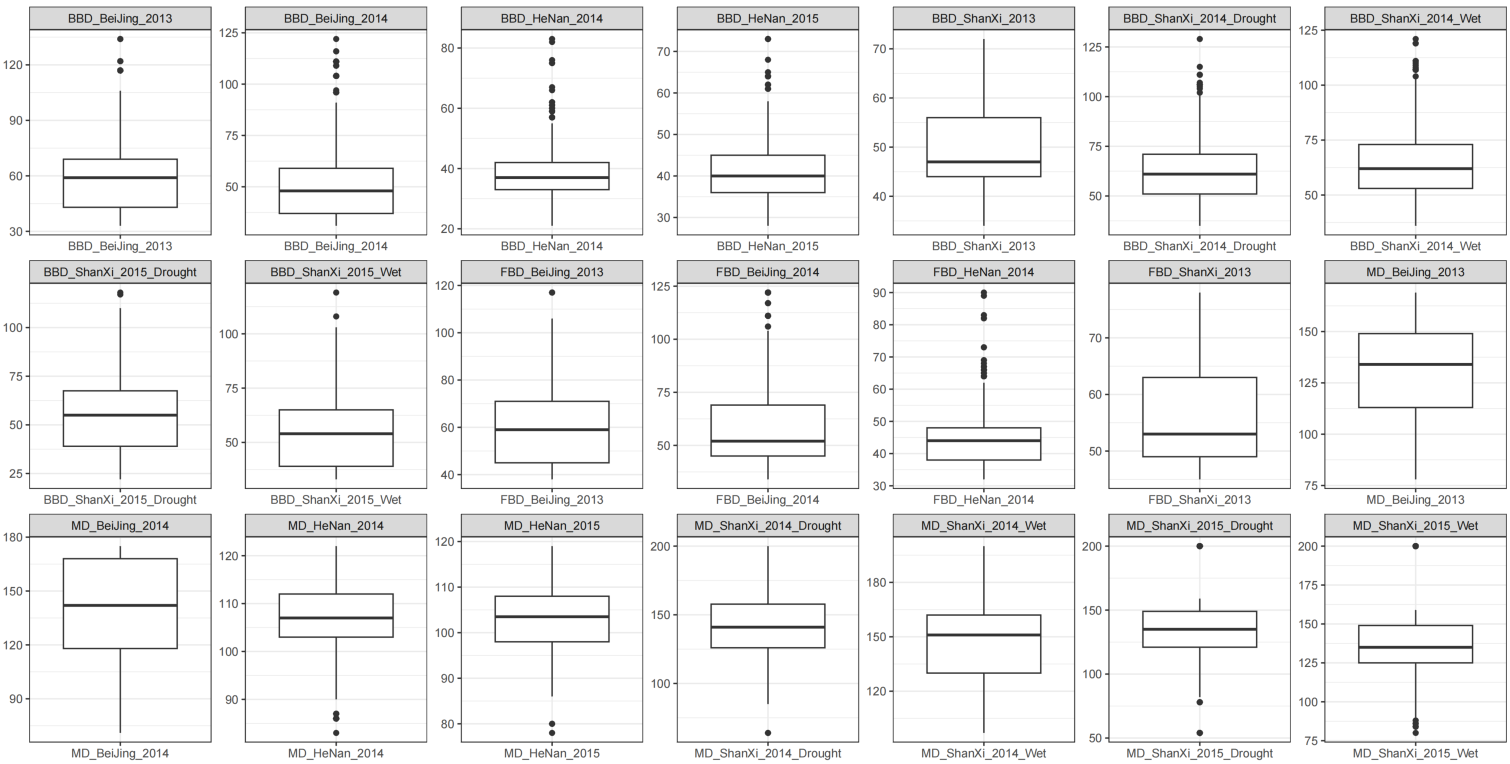


Supplementary Figure 2. Genotype matrix of fourteen genes for a collection of 2,898 soybean accessions. The soybean collection includes wild soybean, landrace, cultivar, colored with purple, pink, and green. The variant of gene are shown on the left. Dark blue, light green, and light blue represent the genotype for the alternative (Alt.) allele, heterozygous, and Zhonghuang13 reference (Ref.) allele, respectively.


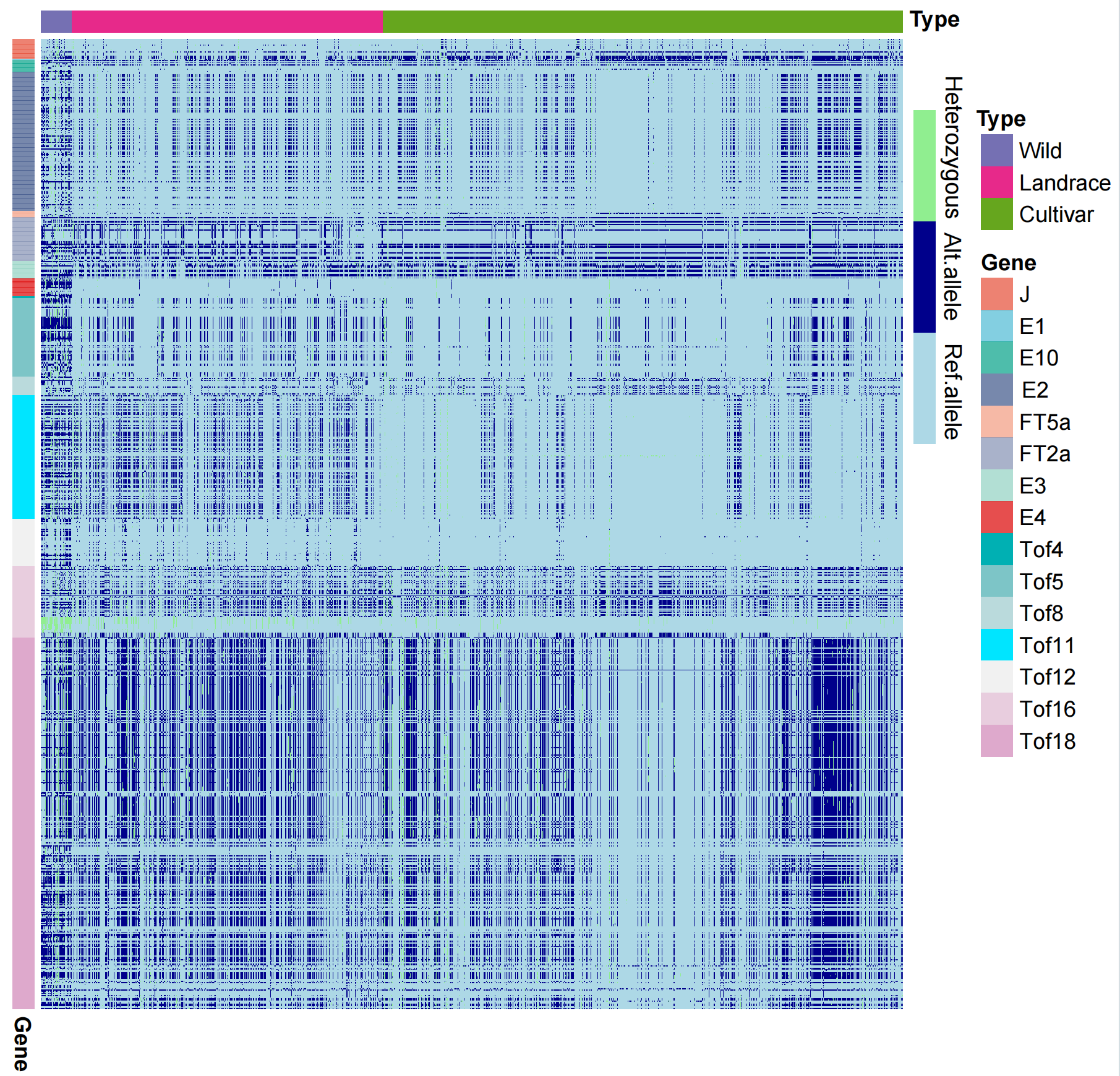


Supplementary Figure 3. The heatmap of pairwise linkage disequilibrium plot with r^2^ for genes in different groups. E1 was not shown due to less variation.


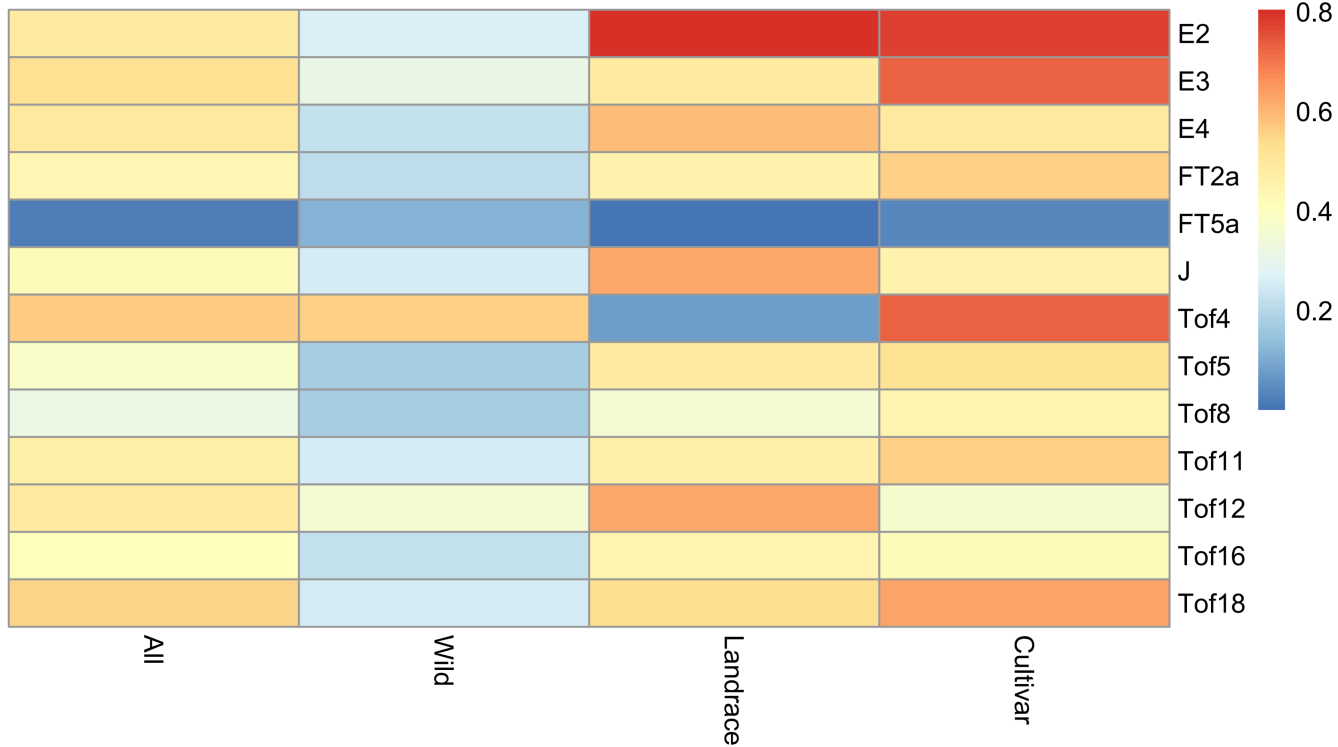


Supplementary Figure 4. Comparative evaluation of predictive capacity for beginning bloom date, full bloom date, and pod maturity date. The predictions for beginning bloom date, full bloom date, and pod maturity date are performed based on the genome-wide SNPs (Scenario 1), and FT SNPs (Scenario 2). The prediction accuracies are measured by the mean values of Pearson correlation coefficient between measure and predicted value with 100 cross-validations (i.e. 20 repetitions of 5-fold cross-validation).


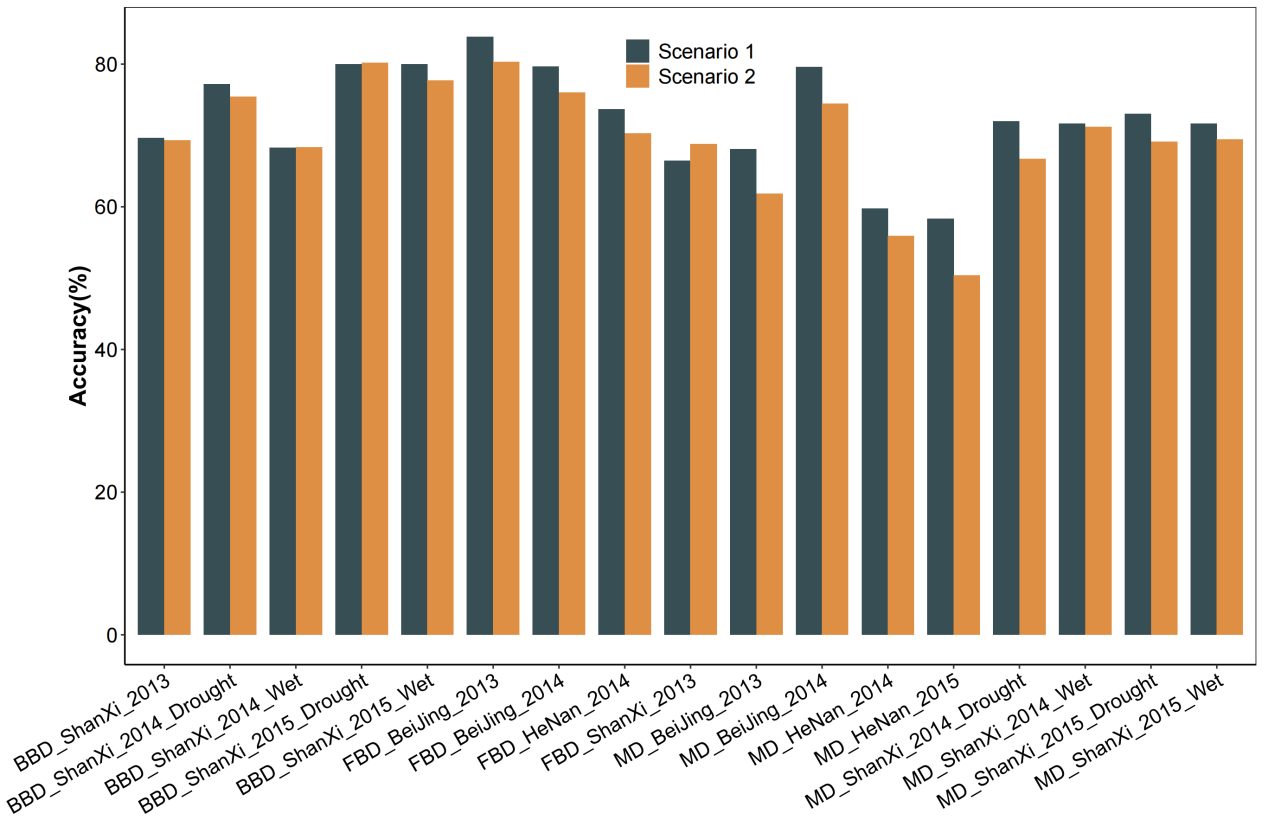


Supplementary Figure 5. GWAS of soybean beginning bloom date in Beijing 2013. Manhattan plots (left) of GWAS for beginning bloom date of 2,898 accessions in Beijing 2013. Pvalues are calculated based on linear mixed model in GWAS and the dashed horizontal line indicates the genome-wide significance threshold (*P*=3.85×10^-8^), which is determined by the Bonferroni test. −log_10_ *P* values are plotted against the position of SNPs on 20 chromosomes. For quantile-quantile plot (right), -log_10_-transformed observed *P* values are plotted against -log_10_-transformed expected *P* values.


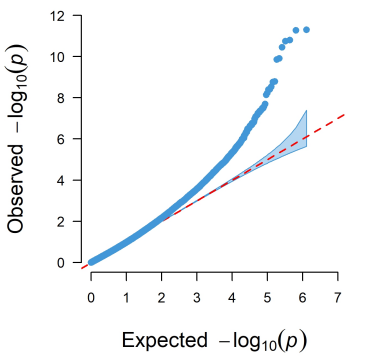

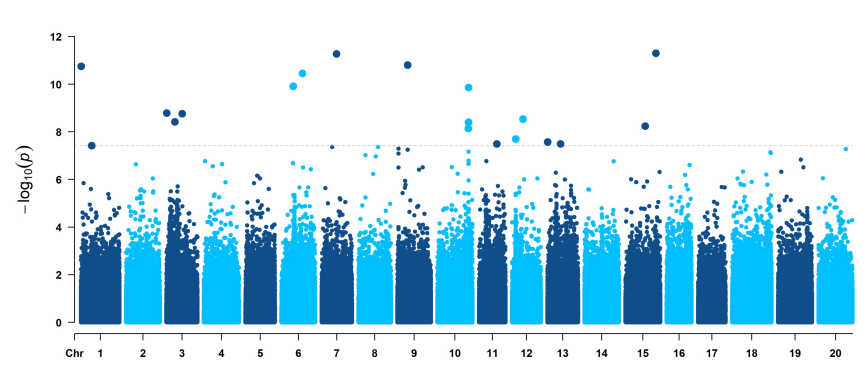


Supplementary Figure 6. GWAS of soybean beginning bloom date in Beijing 2014. Manhattan plots (left) of GWAS for beginning bloom date of 2,898 accessions in Beijing 2014. Pvalues are calculated based on linear mixed model in GWAS and the dashed horizontal line indicates the genome-wide significance threshold (*P*=3.85×10^-8^), which is determined by the Bonferroni test. −log_10_ *P* values are plotted against the position of SNPs on 20 chromosomes. For quantile-quantile plot (right), -log_10_-transformed observed *P* values are plotted against -log_10_-transformed expected *P* values.


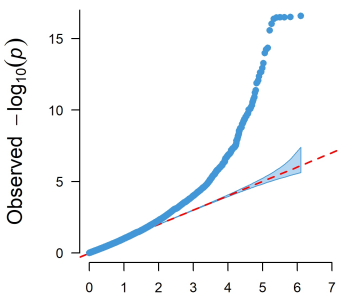

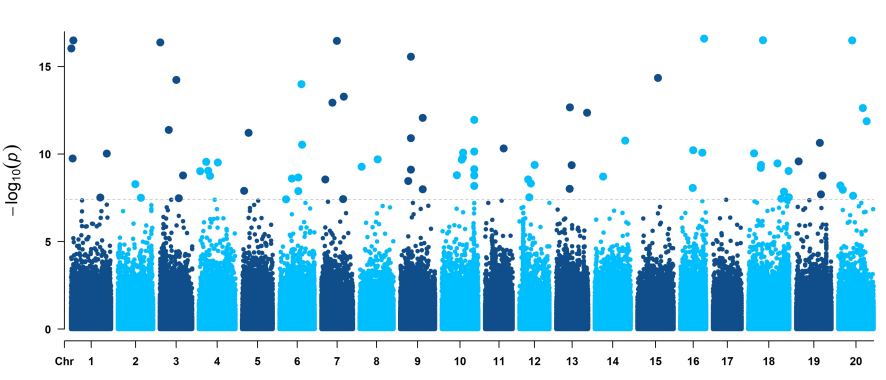


Supplementary Figure 7. GWAS of soybean beginning bloom date in Henan 2014. Manhattan plots (left) of GWAS for beginning bloom date of 2,898 accessions in Henan 2014. Pvalues are calculated based on linear mixed model in GWAS and the dashed horizontal line indicates the genome-wide significance threshold (*P*=3.85×10^-8^), which is determined by the Bonferroni test. −log_10_ *P* values are plotted against the position of SNPs on 20 chromosomes. For quantile-quantile plot (right), -log_10_-transformed observed *P* values are plotted against -log_10_-transformed expected *P* values.


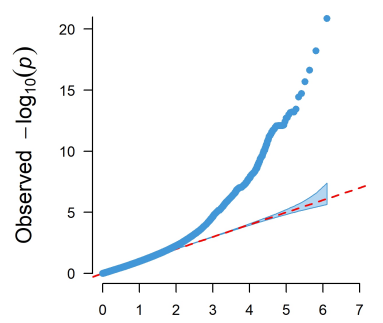

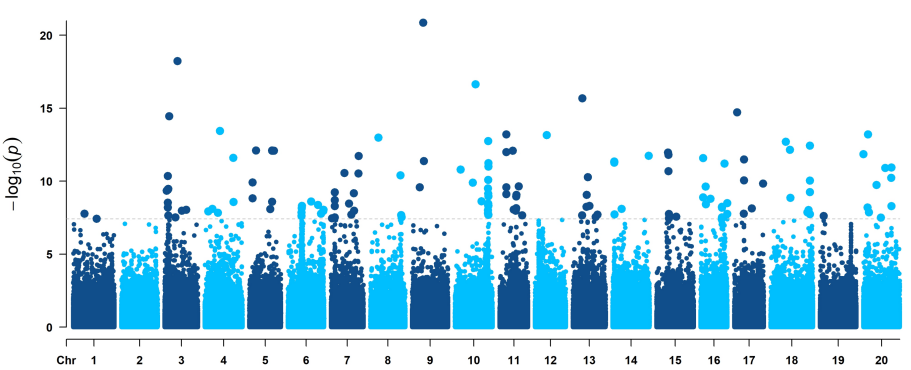


Supplementary Figure 8. GWAS of soybean beginning bloom date in Henan 2015. Manhattan plots (left) of GWAS for beginning bloom date of 2,898 accessions in Henan 2015. Pvalues are calculated based on linear mixed model in GWAS and the dashed horizontal line indicates the genome-wide significance threshold (*P*=3.85×10^-8^), which is determined by the Bonferroni test. −log_10_ *P* values are plotted against the position of SNPs on 20 chromosomes. For quantile-quantile plot (right), -log_10_-transformed observed *P* values are plotted against -log_10_-transformed expected *P* values.


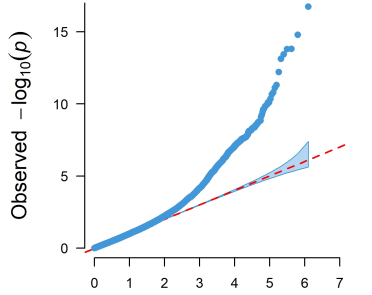

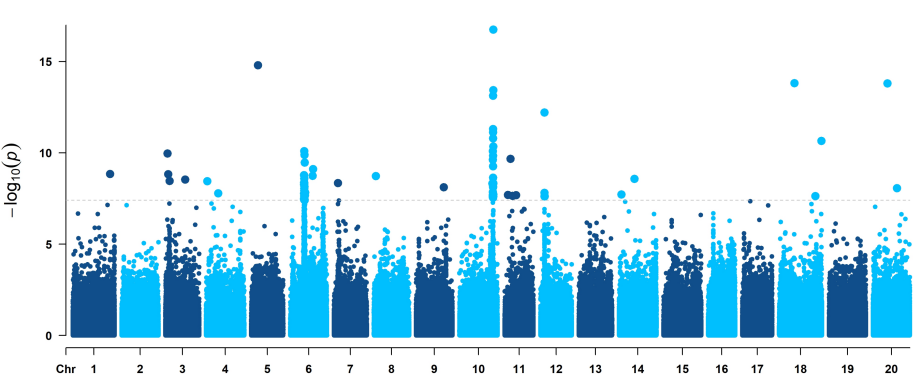


Supplementary Figure 9. GWAS of soybean maturity group in China. Manhattan plots (left) of GWAS for soybean maturity group in China. Pvalues are calculated based on linear mixed model in GWAS and the dashed horizontal line indicates the genome-wide significance threshold (*P*=3.85×10^-8^), which is determined by the Bonferroni test. −log_10_ *P* values are plotted against the position of SNPs on 20 chromosomes. For quantile-quantile plot (right), -log_10_-transformed observed *P* values are plotted against -log_10_-transformed expected *P* values.


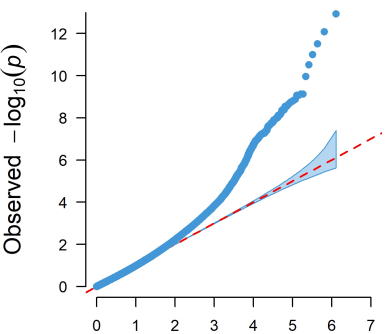

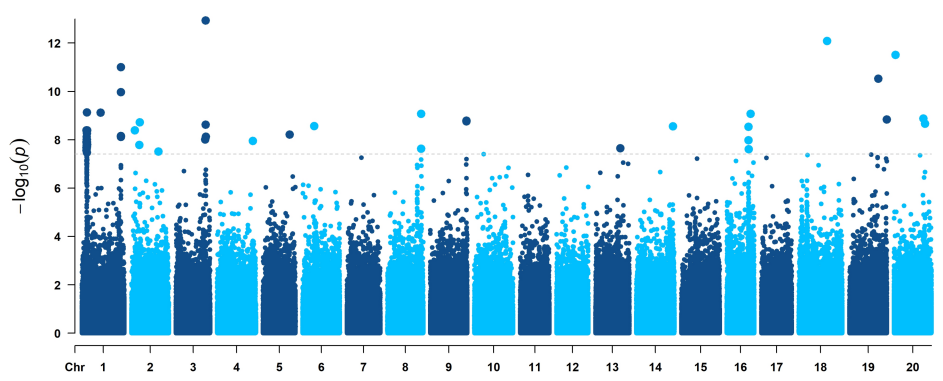


Supplementary Figure 10. The distribution of adjust *p* value via LFMM for 2,898 soybean accessions.


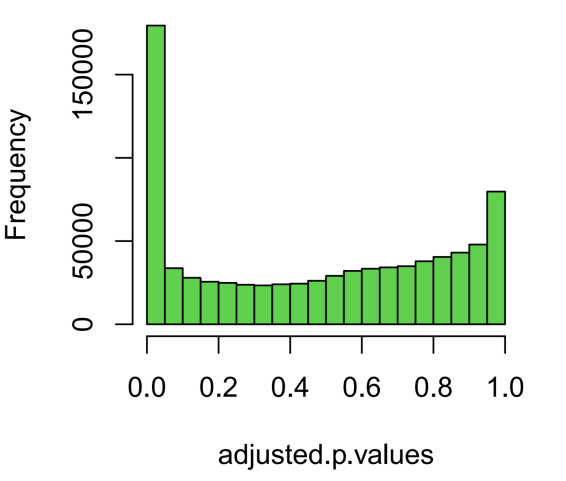


Supplementary Figure 11. Comparison of beginning bloom date, full bloom date, and pod maturity date distribution for samples with different haplotype combinations of seven FT-related genes. Haplotype combinations containing between zero and seven HapA are shown on the x-axis.


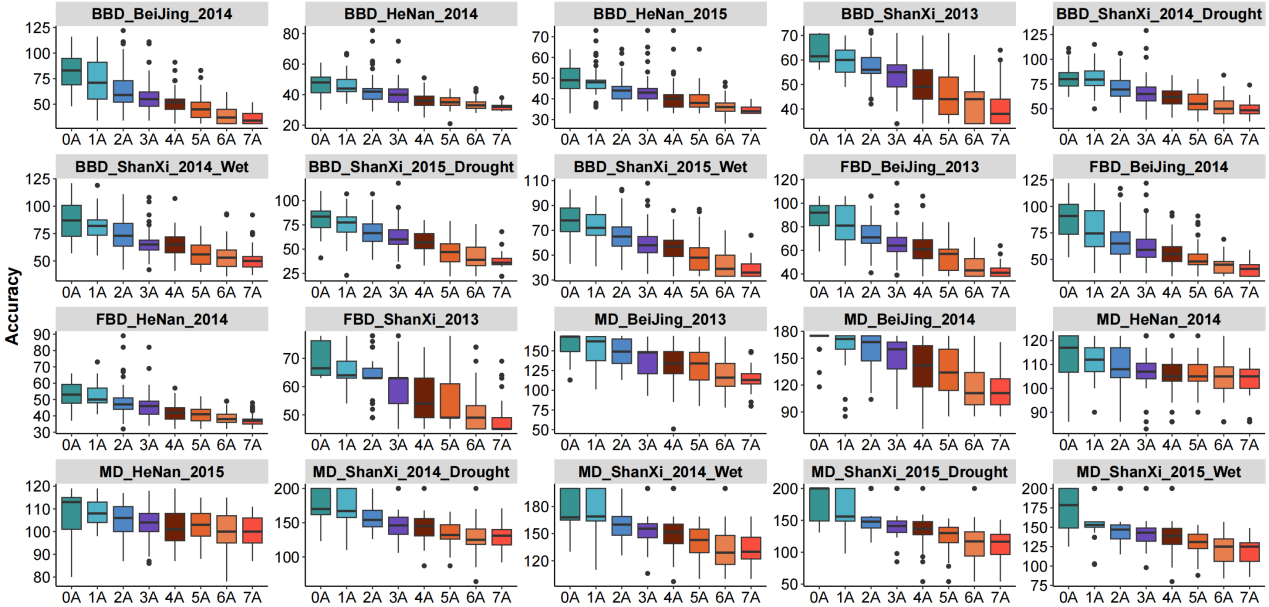

Supplement: Supplementary file 1 — Supplementary Material 1. [file 12870_2024_5382_MOESM1_ESM.docx]
